# All-Dielectric Metalenses for Long-Wavelength Infrared Imaging Applications: A Review

**DOI:** 10.3390/s25123781

**Published:** 2025-06-17

**Authors:** Shinpei Ogawa, Misaki Hanaoka, Manabu Iwakawa, Shoichiro Fukushima, Masaaki Shimatani

**Affiliations:** Advanced Technology R&D Center, Mitsubishi Electric Corporation, 8-1-1 Tsukaguchi-Honmachi, Amagasaki 661-8661, Hyogo, Japan; Hanaoka.Misaki@dh.mitsubishielectric.co.jp (M.H.); Iwakawa.Manabu@cj.mitsubishielectric.co.jp (M.I.); Fukushima.Shoichiro@cb.mitsubishielectric.co.jp (S.F.); Shimatani.Masaaki@bk.mitsubishielectric.co.jp (M.S.)

**Keywords:** metalenses, metasurfaces, metamaterial, infrared sensors, thermal imaging, long-wavelength infrared, achromatic metalens

## Abstract

Infrared imaging has gained considerable attention across diverse fields, including security, surveillance, and environmental monitoring. The need to minimize size, weight, power, and cost (SWaP-C) poses challenges for conventional optical systems like refractive lenses. Metalenses with subwavelength surface patterns have emerged as promising solutions to address these limitations. This review provides a comprehensive analysis of all-dielectric metalenses for long-wavelength infrared (LWIR) imaging applications, a critical spectral region for human detection and analytical applications (such as gas analysis). We examine the limitations of conventional infrared (IR) lens materials and highlight the performance advantages of LWIR metalenses. Key design principles, including chromatic and achromatic lens configurations, are discussed alongside their imaging performance. Additionally, we review advanced functionalities such as polarization control, multifocal capabilities, zoom, and reconfigurability. Theoretical performance limits and trade-offs are analyzed to provide insights into design optimization. We identify future challenges related to advanced design methods and fabrication techniques. LWIR metalenses can be expected to overcome the shortcomings of conventional LWIR lenses owing to meta-optics technologies, to achieve SWaP-C and advanced functionalities that cannot be achieved by conventional LWIR lenses. This review will guide researchers in academia and industry to develop LWIR metalenses to advance IR imaging technologies.

## 1. Introduction

Infrared (IR) imaging has attracted considerable attention owing to its increasing demand across various fields, including security, surveillance, search and rescue, firefighting, traffic systems, medical imaging, contactless temperature measurement, and equipment maintenance [1,2]. IR imaging typically targets two primary wavelength bands: the mid-wavelength IR (MWIR) band, spanning 3–5 μm, and the long-wavelength IR (LWIR) band, covering 8–14 μm. MWIR imaging is suited for detecting high-temperature objects, such as fires, while LWIR imaging is ideal for observing lower-temperature targets, including the human body. Consequently, LWIR imaging is more commonly used to meet day-to-day requirements. Therefore, this review focuses on LWIR. To detect these two bands, remarkable IR photodetector advances have been achieved. IR photodetectors are typically classified into two categories, as follows: thermal (uncooled) detectors [3], such as bolometers [4], thermopiles [5], and pyroelectric sensors [6]; and quantum-type detectors [7], including HgCdTe [8], type-II superlattices [9], and two-dimensional materials [10,11]. Both detector types are advancing rapidly and are increasingly being commercialized. However, IR cameras must also minimize size, weight, power consumption, and cost (SWaP-C), attributes that conventional optical systems struggle to achieve. The performance and cost of IR imaging cameras depend on the photodetectors and (most importantly) lenses. Notably, in the MWIR and LWIR wavelength regions, there are no lens materials that match the performance and cost-effectiveness of those used in the visible or near-IR (NIR) regions.

### 1.1. Conventional LWIR Lens Materials

**Figure 1:**
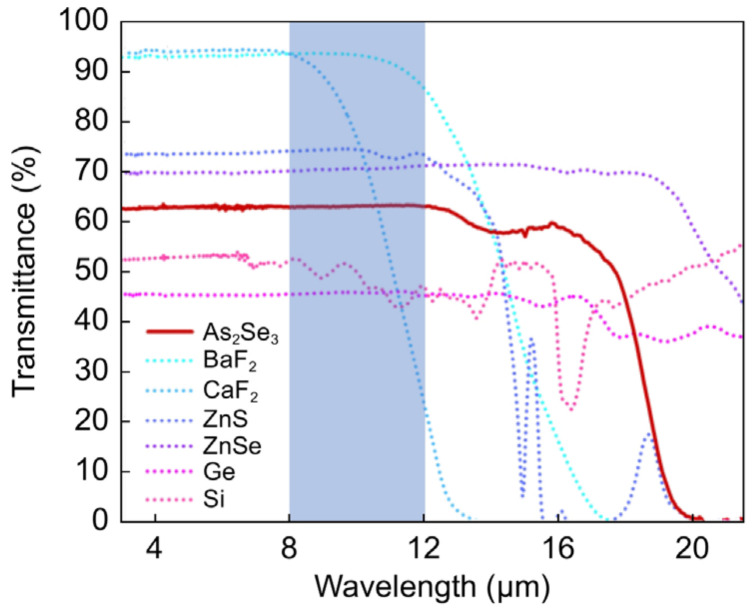
Transmission spectra of conventional lens materials for long-wavelength infrared (LWIR). The thickness for all materials is 1 mm, except for As_2_Se_3_, which has a thickness of 1.5 mm. Adapted from [12] under CC BY 4.0.

Figure 1 presents the transmission spectra of conventional LWIR lens materials, including arsenic triselenide (As_2_Se_3_), which belongs to the class of chalcogenide glasses, barium fluoride (BaF_2_), calcium fluoride (CaF2), zinc sulfur (ZnS), zinc selenide (ZnSe), germanium (Ge), and silicon (Si) [12]. During transmittance measurements, surface reflection was observed for each material. Table 1 and Table 2, respectively, present a comparison of these materials from the perspectives of material properties and product characteristics.

As presented in Table 1 and Table 2, conventional LWIR lens materials exhibit various advantages and disadvantages. For example, Ge is limited by its high cost, while Si experiences higher intrinsic loss than Ge. Chalcogenide glasses have higher thermal expansion coefficients and dn/dT values compared with those of Ge and Si, leading to focus shifts with temperature fluctuations. ZnS and CaF_2_ offer lower environmental resistance than Ge and Si, whereas ZnSe and BaF_2_ present toxicity concerns. Although numerous studies, particularly on chalcogenide glasses, are being conducted to overcome these challenges [18,19], Ge-based LWIR lenses remain the most commonly used for high-performance LWIR imaging applications owing to their high transmittance and productivity. Conversely, Si-based LWIR lenses are used only when their thicknesses can be sufficiently reduced to mitigate the intrinsic loss associated with Si.

### 1.2. Advantages and Challenges of LWIR Metalenses

As discussed in the previous section, all conventional LWIR lens materials are associated with inherent challenges. However, if Si-based LWIR metalenses can overcome the challenge associated with transmission enhancement, they could represent the optimal solution for realizing ideal LWIR lenses. This potential is largely attributed to the maturity of Si fabrication technologies and the compatibility of Si-based LWIR metalenses with complementary metal–oxide–semiconductor processes. We believe that metalens technology can address these challenges, as metalenses can precisely control light through their surface structures, surpassing material-restricted optical properties by leveraging the metamaterial concept [25,26]. However, recent studies on metalenses have primarily focused on the visible and NIR wavelength regions. Metalenses in these regions can manipulate photons through single-layer surface structures with subwavelength dimensions, achieving wafer-level thickness, lightweight form factors, ease of integration, and multifunctionality [27,28,29,30,31]. The advantages of metalenses in the visible and NIR wavelength regions compared with those of diffractive lenses remain controversial [32,33]. However, if similar advancements can be achieved in the LWIR wavelength region, Si-based LWIR metalenses could represent the optimal solution for IR imaging applications, leading to superior performance, high productivity, and lower costs. Additionally, the multifunctionality of metalenses, such as wavelength and polarization selectivity [34], has the potential to expand IR imaging applications significantly.

This review focuses on all-dielectric LWIR metalenses for LWIR imaging applications, considering theoretical limitations and associated challenges, as LWIR imaging is more prevalent across various application fields. The advantages of LWIR metalenses are discussed, and future challenges are highlighted. This review aims to provide an overview of the fundamental physics underlying metalenses and the current state of the art in LWIR metalens applications to support the development of ideal LWIR lenses.

## 2. Theoretical Background of Metalenses

**Figure 2:**
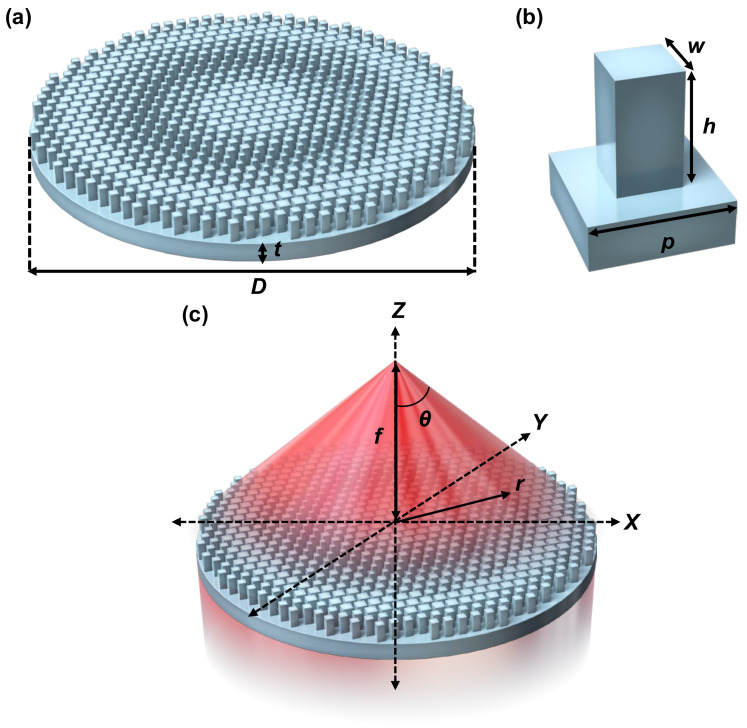
(**a**) Schematic of a metalens, (**b**) a unit cell, referred to as a meta-atom, and (**c**) schematic of focusing by a metalens.

Figure 2a, Figure 2b, and Figure 2c, respectively, illustrate the schematics of a metalens and its unit cell, referred to as a meta-atom, and the schematic depicting the process of metalens focusing.

The diameter and focal length of the metalens are defined as *D* and *f*, respectively. The half-angle of the maximum cone of light exiting the metalens is denoted by θ. The period of the meta-atom is represented as *p*. The height and width of the pillar on the meta-atom are defined as *h* and *w*, respectively. The thickness of the substrate is represented as *t*. The total thickness of the metalens is approximately equal to *t* + *h* ≅ *t* because *t* is significantly greater than *h* (*t* >> *h*). This review adopts a polar coordinate system (Figure 2c), where the position is defined by *r*. The refractive index of the meta-atom material is defined as *n_d_*. Although complementary hole arrays can be used as alternatives to pillar arrays in metalenses, achieving the required phase shift through hole depth is challenging. This difficulty arises from the low refractive index of materials within the holes, such as air [35], as discussed in the following section.

### 2.1. Basic Theory

A hyperboloidal phase profile with a normal incidence angle for focusing a spot after transmission through a metal surface is expressed as [28],

(1) $$\varphi \left(r,\omega \right)\;=\frac{n_{b}\omega }{c}\left(f-\sqrt{{r^{2}+f}^{2}}\right)=\frac{{2\pi n}_{b}}{\lambda }\left(f-\sqrt{{r^{2}+f}^{2}}\right),$$

where *n_b_* represents the refractive index of the background medium, *c* is the speed of light in a vacuum, *ω* is the circular frequency, and *λ* is the wavelength. Equation (1) shows that the required phase profile can be determined by a spatial factor, such as the focal length (*f*) and the specific position (*r*) in the metalens, according to the operating wavelength. Equation (1) is for the normal incident angle (*θ_i_* = 0°). For any arbitrary *θ_i_*, the aforementioned phase profile is modified according to *θ_i_* and is ideally expressed as [28,36]:

(2) $$\varphi \left(r,\lambda ,\;\theta_{i}\right)\;=-\frac{{2\pi n}_{b}}{\lambda }\left(rsin\theta_{i}+\sqrt{{{\left(r-ftan\theta_{i}\right)}^{2}+f}^{2}}-\frac{f}{cos\theta_{i}}\right).$$

However, Equation (1) is commonly used for the design of conventional metalenses [28].

The phase profiles of different metalenses with a single focal point can be combined into one phase profile. The combined phase profile of multifocal metalenses that have *N* focal points on the same focal plane can be expressed as [37,38]:

(3) $$\phi \left(x,y\right)=arg\sum_{j=1}^{N}e^{i\varphi_{j}(x,y)},$$

(4) $$\varphi_{j}\left(x,y\right)\;=\frac{{2\pi n}_{b}}{\lambda_{0}}\left(f_{Dj}-\sqrt{f^{2}+{\left(x-x_{j}\right)}^{2}+{\left(y-y_{j}\right)}^{2}}\right),$$

(5) $$f_{Dj}=\sqrt{f^{2}+r^{2},}$$

(6) $$r=\sqrt{{x_{j}}^{2}+{y_{j}}^{2}}.$$

Equations (3) and (4) are derived from the single focus metalens by expanding Equations (1) and (2); these equations express the phase profile as a function of the focal point and use the coordinate (*x_j_*, *y_j_*) at wavelength λ_0_.

### 2.2. Design Parameter of Meta-Atoms

A meta-atom is designed to satisfy Equations (1)–(6). The required 2π phase delay can be achieved primarily through three mechanisms, as follows: (i) resonance, (ii) propagating phase, and (iii) geometric phase. (i) The resonance phase can be induced by several types of resonances, including the plasmonic [39,40], Mie [41,42], and Fabry–Perót resonances [43]. (ii) The propagating phase induces a phase shift by controlling the propagation of light along the *h*-direction (*z*-axis direction) of the meta-atom in Figure 2b,c, as described by the following equations [27,28]:

(7) $$\varphi_{j}\left(x,y,\lambda \right)\;=\frac{2\pi }{\lambda }n_{eff}\left(x,y,\lambda \right)h,$$

(8) $$h=\frac{\lambda }{n_{d}-1},$$

where *n_eff_* is the local effective refractive index of the meta-atoms and is defined as a weighted average of the refractive indices of the different regions within a structure, such as the meta-atom material (e.g., silicon) and the surrounding medium (e.g., air). (iii) The geometric phase induces a phase shift through the rotational orientation of anisotropic meta-atoms. This mechanism is related to points on the Poincaré sphere [44] and is also known as the Pancharatnam–Berry (PB) phase [45,46].

**Figure 3:**
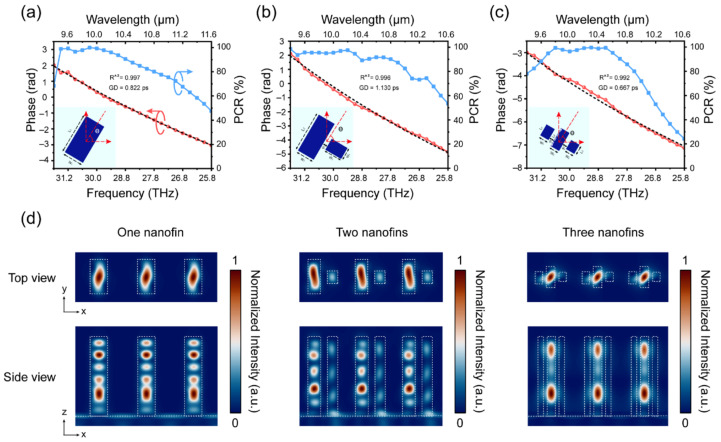
Calculated phase shifts as a function of the wavelength and frequency for meta-atoms comprising (**a**) one, (**b**) two, and (**c**) three rods. (**d**) Magnetic field distribution for the corresponding meta-atoms. Adapted from [47] under CC BY 4.0.

Among these strategies, the resonance phase (i) operates within a narrow bandwidth, which can lead to errors when dealing with large phase gradients and consequently degrade lens performance. Therefore, recent design strategies primarily adopted the propagating phase (ii) and/or the geometric phase (iii), particularly when designing achromatic metalenses. Figure 3a–d illustrate the examples of the phase shifts and the normalized magnetic field intensity distributions for three types of meta-atoms comprising a single rod, two rods, and three rods, respectively [47].

Figure 3 shows that phase delays occur during the propagation of light along the rods in the *h*-direction (*z*-direction in Figure 3d). As shown in Figure 3a–c, the phases (red and black dotted lines) are almost linear with respect to the frequency within the operating bandwidth for all meta-atom structures. The group delays for one, two, and three nanofins are 0.82 ps, 1.13 ps, and 0.67 ps, respectively. Additionally, the geometric phase changes according to the polarization conversion ratio (blue lines), which is based on the PB phase. Moreover, Figure 3d shows that a larger phase compensation is achieved owing to the waveguide-like mode in the single nanofin case and the coupling between propagating modes in the cases of two and three nanofins. This type of dispersion engineering, based on the propagating phase (ii) and/or the geometric phase (iii), is key to achieving ideal metalenses, regardless of the operating wavelength region. Another important design parameter of lenses is the numerical aperture (*NA*). *NA* is defined as the product of the refractive index of the image space and the sine of the cone half-angle of light. Therefore, *NA* defines the resolution of lenses. According to diffraction theory, the full width at half maximum (*FWHM*) of the focal spot size is determined by the *NA*. The relationships between *NA* and *FWHM* are described by the following equations [28]:

(9) $$\mathrm{NA}=n_{d}sin\theta ,$$

(10) $$\mathrm{FWHM}=\frac{\lambda }{2NA}.$$

However, metalenses possess a spatially discrete structure composed of meta-atom arrays, where the distance between the centers of adjacent meta-atoms is defined as *p*. This spatial discretization *p* limits the maximum achievable *NA* based on the Nyquist−Shannon sampling theorem in the spatial domain [48]. To satisfy the sampling criterion, the following condition must be met:

(11) $$p\leq \frac{\lambda }{2NA}.$$

Equation (11) shows that the meta-atom size of *p* should be reduced to obtain metalenses with higher *NA* values. It should be noted that the fabrication limit, such as the photolithography resolution, should be considered to determine *p* in conjunction with the desired *NA*.

The fundamental design parameters of meta-atoms, such as *p*, *h*, and *d*, play crucial roles in the design of the phase profile required for metalens. The relation between these parameters and lens performance, such as *NA*, *FWHM*, and resolution, provides essential insights for developing metalenses regardless of their operating wavelengths.

## 3. Chromatic LWIR Metalenses

**Figure 4:**
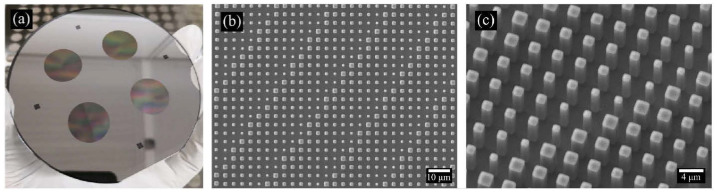
Simple Si-wafer-based LWIR metalenses: (**a**) optical image, (**b**) scanning electron microscopy (SEM) image, and (**c**) magnified view of the Si-wafer-based LWIR metalenses used in the study. Adapted with permission from [50]. © Optical Society of America.

To our knowledge, Fan et al. [49] were the first to demonstrate the development of LWIR metalenses, while the study by Huang et al. [50] presents the first LWIR images obtained using LWIR metalenses. Both studies employed simple Si-based LWIR metalenses designed based on Equation (1). Figure 4a–c, respectively, shows the optical image, scanning electron microscopy (SEM) image, and a magnified view of the Si-wafer-based LWIR metalenses used in these studies. As shown in Figure 4, all-Si-based metalenses can be fabricated using a 4-inch Si wafer, where the surface metalens pattern was successfully formed by simple photolithography and dry-etching techniques.

**Figure 5:**
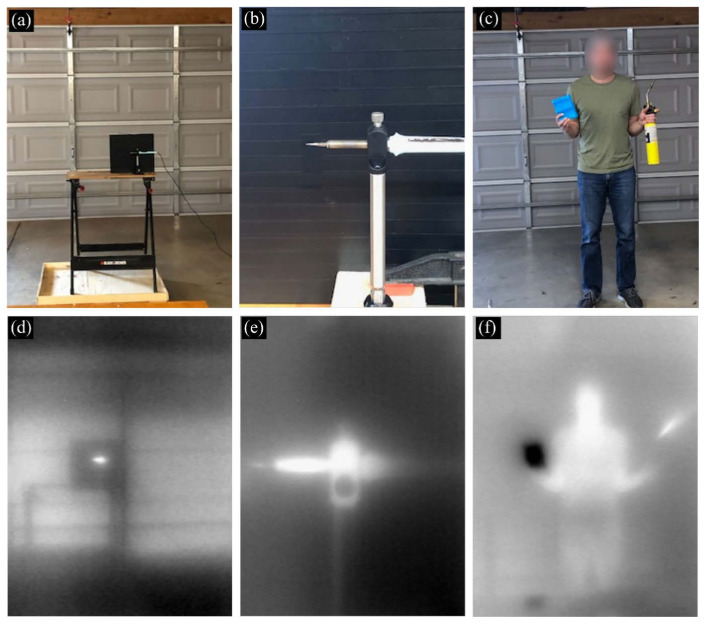
LWIR images obtained using the Si-wafer-based LWIR metalens: (**a**) hot soldering iron positioned in front of a steel plate covered by black tape with an emissivity of 0.95; (**b**) magnified view of the soldering iron on a metal holder, as shown in (**a**); (**c**) person holding an ice pack (left) and a hot blowtorch (right); (**d**), (**e**), and (**f**) corresponding LWIR images of scenes in (**a**), (**b**), and (**c**), respectively. Adapted with permission from [50]. © Optical Society of America.

Figure 5 presents the LWIR images obtained using the LWIR camera equipped with the LWIR metalens shown in Figure 4.

As shown in Figure 5, although the metalenses used exhibit strong chromatic aberration, the temperature differences of the objects can still be distinguished under ambient light conditions. In particular, as shown in Figure 5c,f, the temperature difference between the ice pack and hot blowtorch was clearly distinguishable. This is because MWIR and LWIR radiation emitted by the objects follows Planck’s law, exhibiting broad emission peaks corresponding to their temperatures. Consequently, chromatic aberration is less pronounced in the LWIR region compared with the visible or NIR regions. This characteristic represents a substantial advantage for LWIR imaging using LWIR metalenses. Moreover, Si-based LWIR metalenses can be easily fabricated using conventional photolithography and deep-reactive-ion etching methods. This ease of fabrication is attributed to the structural parameters of the meta-atoms that have sizes similar to the primary operating wavelength of approximately 10 μm [51,52]. At this scale, expensive and complex fabrication techniques, such as electron beam lithography and extreme ultraviolet lithography, are not required. In contrast, metalenses designed for visible or NIR wavelengths typically necessitate these advanced systems. To the best of our knowledge, the integration of a chromatic metalens with an aperture of 5 cm in a thermographic camera has already been demonstrated [53]. This study provides strong evidence that simple LWIR metalenses are suitable for practical applications.

## 4. Achromatic LWIR Metalenses

Conventional refractive lenses also suffer from chromatic aberration owing to their material dispersion. However, diffractive elements, such as meta-atom-based metalenses and gratings, exhibit even stronger chromatic aberration [54]. Consequently, correcting chromatic aberration is a primary challenge for metalenses, regardless of the operating wavelength. This challenge is particularly critical in the LWIR region because its target wavelength range is 8–14 μm, which is broader than other spectral regions. Therefore, effective chromatic aberration elimination is essential for LWIR metalenses. The design principle of achromatic metalenses can be described using the Taylor expansion of the phase profile in Equation (1) [55],

(12) $$\varphi \left(r,\omega \right)\;=\varphi \left(r,\omega_{d}\right)+{\left.\frac{\partial \left(r,\omega \right)}{\partial \omega }\right|}_{\omega_{d}}\left(\omega -\omega_{d}\right)+{\left.\frac{\partial^{2}\left(r,\omega \right)}{2\partial \omega^{2}}\right|}_{\omega_{d}}{\left(\omega -\omega_{d}\right)}^{2}+\ldots .$$

The first, second, and third terms on the right-hand side of Equation (12) correspond to the relative phase, relative group delay (*RGD*), and GD dispersion, respectively. The relative phase is the phase difference between light waves propagating through metalenses. *RGD* is the time delay difference experienced by different frequency components. GD dispersion describes the extent to which the group delay varies with frequency. Equation (12) indicates that LWIR metalenses must satisfy all three conditions to achieve achromatic focusing within a target achromatic bandwidth (Δ*ω*) around *ω_d_*. Δ*ω* is defined as the difference between the smallest and largest frequencies over which the desired operation can be performed. In this context, achromatism can be realized over Δ*ω*. The wavefront at *ω_d_* can be designed using Equation (1). The GD and GD dispersion describe the chromatic focal length shift of metalenses [55]. The *RGD* is expressed as follows:

(13) $$\mathrm{RGD}\left(r\right)\;=\frac{\partial \left(r,\omega \right)}{\partial \omega }=\frac{n_{b}}{c}\left(f-\sqrt{{r^{2}+f}^{2}}\right).$$

Accordingly, the GD dispersion ($\frac{\partial^{2}\left(r,\omega \right)}{2\partial \omega^{2}}$) is zero because *RGD* is not a function of *ω* (see Equation (13)). Two primary methods have been proposed to achieve this design principle. The first involves the modification of the geometric parameters of waveguide-type meta-atoms. The second combines adjustments to both the PB phase and the geometric parameters of waveguide-type meta-atoms. However, there is a fundamental limit to the achievable Δ*ω* [56]. This limit is governed by the time delay (Δ*t*) of the transmitted light between the center and edge of the metalens and is expressed using a dimensionless quantity κ as follows [56]:

(14) $$\Delta \omega \leq \frac{\kappa }{\Delta t},$$

where κ expresses an upper bound for Δ*t* experienced by the signal and Δ*ω*, which is described as the delay–bandwidth or time–bandwidth product [56]. The maximum time delay (Δ*t_max_*) is given as follows [56]:

(15) $$\Delta t_{\max}\;=\frac{fn_{b}}{c}\left(\sqrt{1+{\left(\frac{D/2}{f}\right)}^{2}}-1\right).$$

Thus, the limit of Δ*ω* of the metalenses can be described in terms of their *NA* and geometric parameters as follows [56]:

(16) $$\Delta \omega \leq \frac{\kappa c}{fn_{b}\left(\sqrt{1+{\left(\frac{D/2}{f}\right)}^{2}}-1\right)}=\frac{\kappa c\sqrt{1-{\left(\frac{NA}{n_{b}}\right)}^{2}}}{fn_{b}\left(1-\sqrt{1-{\left(\frac{NA}{n_{b}}\right)}^{2}}\right)}.$$

As defined previously, “*c*” is the speed of light. A one-dimensional, lossless dielectric pillar can be considered a waveguide. Therefore, the upper bound of κ in waveguide-type metalenses is given by [56,57]:

(17) $$\kappa =2\pi \frac{h}{\lambda }\left(n_{max}-n_{min}\right).$$

where *n_max_* and *n_min_* are the maximum and minimum effective refractive indices of metalenses. For further details on resonant or other types of meta-atom-based metalenses, please refer to Reference [56].

**Figure 6:**
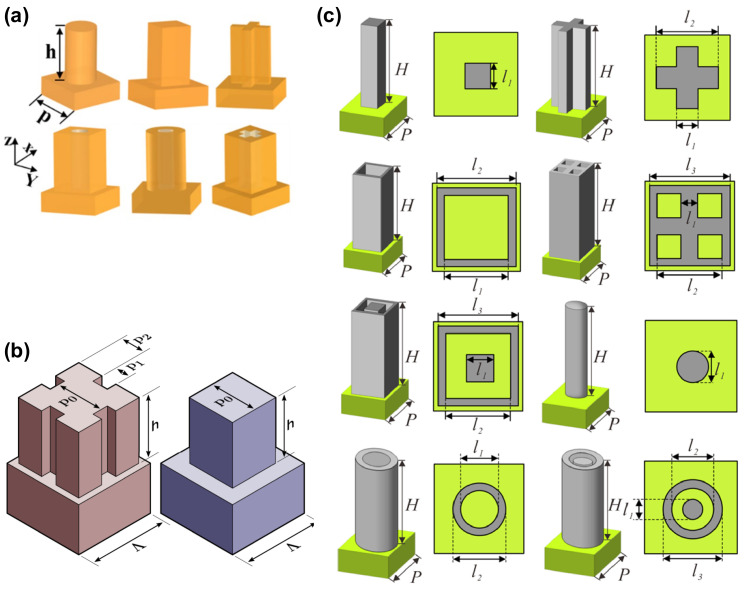
Various building blocks of meta-atoms for achromatic LWIR metalenses: (**a**) six types of meta-atoms, (**b**) complex (**left**) and simple (**right**) scatterers, and (**c**) eight types of meta-atoms. Subfigure (**a**) is adapted from [67] under CC BY 4.0; subfigure (**b**) is adapted from reference [68] under CC BY 4.0, and subfigure (**c**) is adapted with permission from [69]. © Optical Society of America.

The primary strategy used to eliminate chromatic aberration involves the use of multiple meta-atoms within the unit cell, either arranged in-plane or vertically stacked, to construct metasurfaces capable of supporting multiple wavelengths [44,54,58,59]. Recently, a rapid increase in the development of achromatic LWIR metalenses has been reported [47,60,61,62,63,64,65,66,67,68]. Among these previous studies, the widest wavelength range achieved was 8–12 μm [66,67,68]. Figure 6 shows examples of the multiple meta-atoms used in achromatic LWIR metalenses. These structures typically exhibit four-fold symmetry to achieve polarization insensitivity, and more complex shapes have been introduced to induce multiple wavelength scattering.

**Figure 7:**
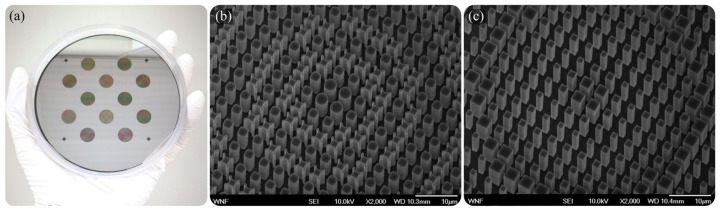
(**a**) Photograph of achromatic LWIR metalenses formed in a Si wafer. SEM images of (**b**) achromatic and (**c**) chromatic LWIR metalenses. Adapted from [68] under CC BY 4.0.

Figure 7 illustrates the fabricated achromatic and chromatic LWIR metalenses on Si wafers [68].

**Figure 8:**
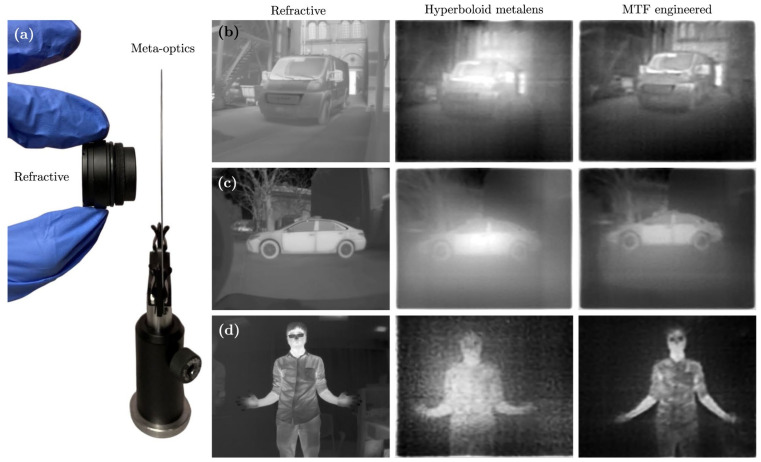
(**a**) Comparison of metalenses with conventional refractive lenses. LWIR images obtained using (**b**) conventional refractive lenses, (**c**) chromatic LWIR metalenses, and (**d**) achromatic LWIR metalenses. Adapted from [68] under CC BY 4.0.

Figure 8 shows a comparison of the LWIR images obtained using (a) conventional lenses, (b) chromatic metalenses, and (c) achromatic metalenses for a truck parked outdoors, a car parked outdoors, and a person with hands stretched out indoors, respectively. All images were obtained under ambient daylight conditions. In all cases, the difference between chromatic and achromatic metalenses is clearly distinguishable. Particularly, the shirt patterns can be recognized because the small thermal differences can be distinguished owing to the achromatism effect. However, the IR images obtained by the conventional refractive lenses are clearer than those obtained by achromatic metalenses. This difference may be overcome using various advanced design methods (as discussed in Section 8). The transmittance of the metalenses can be improved using an antireflection coating. Figure 6 and Figure 8 indicate that high-resolution LWIR imaging requires an achromatic metalens.

## 5. High *NA*

High *NA* values are advantageous in the LWIR region in applications. Small light–matter interaction volumes and broader angular collection are required in high-resolution imaging and spectroscopy [70]. Achieving a high *NA* involves the precise alignment of a sequence of compound lenses or aspherical lenses. In contrast, a single metalens can achieve a high *NA* because metalenses with hyperbolic phase profiles are inherently free from spherical aberrations.

The *NA* is determined by *p* of the meta-atoms, as described by Equation (11), and can be expressed as follows:

(18) $$NA\leq \;\frac{\lambda }{2p}.$$

As evident, a smaller *p* is desirable for a high *NA*. However, reducing *p* limits the variation in the lateral dimension of meta-atoms, thereby decreasing the achievable phase delay. To compensate for this reduction and achieve a high *NA*, a higher refractive index and/or a larger aspect ratio (*h*/*w*) are required. To the best of our knowledge, an achromatic LWIR metalens operating in the 9–11 μm range with the highest *NA* value of 0.79 can be theoretically achieved using a high-aspect ratio of 15 [64].

Notably, there is a fundamental trade-off between focusing efficiency and high *NA* values. As the phase gradient increases and the phase discretization level decreases owing to the limited *p*, the diffracted efficiency at the edges of the metalens is reduced. This reduction results in reduced focusing efficiencies [28]. Addressing this trade-off requires advanced design methodologies, which will be discussed in Section 8.

## 6. Wide Field of View

Wide fields of view (FOVs) are essential for applications such as image projection, landscape imaging, and security surveillance. Conventionally, IR optical systems rely on compound lenses, such as fisheye lenses, to suppress coma aberrations [71]. However, these systems increase the overall cost and size of IR camera setups.

**Figure 9:**
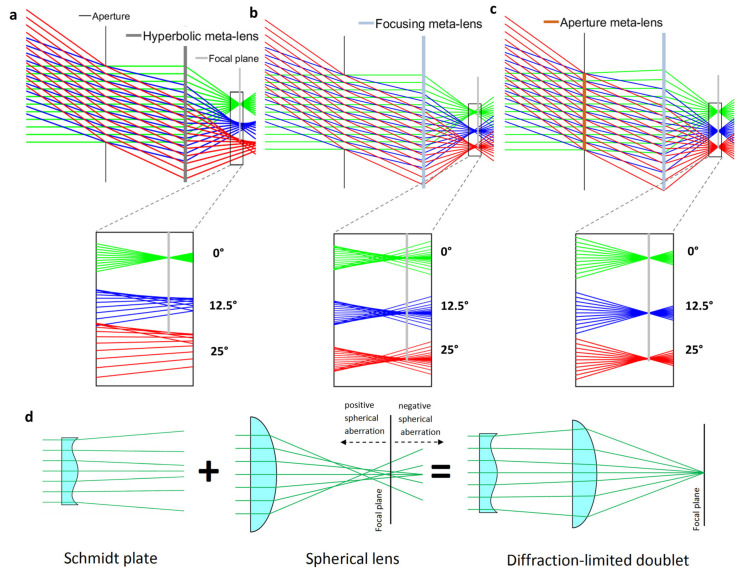
Aberration correction concept of metalenses and comparison with conventional lenses for wide field-of-view applications. (**a**) Singlet conventional metalens with an aperture setup, exhibiting large aberrations at oblique incidence. (**b**) Focusing metalens with and without the aperture metalenses. (**c**) Doublet metalens consisting of an aperture and focusing metalenses. (**d**) Comparison with conventional refractive lenses. Adapted with permission from [73]. Copyright 2017 American Chemical Society.

If metalenses can manipulate IR light at arbitrary incident angles by incorporating incident angle-dependent phase control, as described in Equation (2), a wide FOV can be achieved using a flat metalens. However, conventional metalens designs typically assume incident angle independence. To overcome this challenge, three types of metalens structures have been proposed, as follows: (i) singlet aplanatic, (ii) singlet with aperture (Chevalier landscape), and (iii) doublet aplanatic metalenses [30]. Among these, the singlet aplanatic metalens offers fewer degrees of freedom compared with the other two types. Consequently, the singlet with aperture and doublet aplanatic metalens designs are primarily considered. Pioneering studies (specifically in (ii) [72] and (iii) [73]) on these designs were initially conducted in the visible wavelength region. Figure 9 illustrates the concept of the doublet aplanatic metalens.

As shown in Figure 9a, the small aperture formed in front of the metalens can separate normal and oblique incident light. As a result, incident light at different angles can interact with different parts of the metalens, thus allowing incident angle-dependent control of the phase profile in the metalenses to suppress aberrations [30]. This metalens type corresponds to a focusing metalens, as shown in Figure 9b. As shown in Figure 9c, the doublet metalens configuration can correct the positive and negative spherical aberration. It can be analogically described as shown in Figure 9d. The effective focal length can be increased by diverging the marginal rays generated by the Schmidt plate. Conversely, the effective focal length can be decreased by converging the chief ray. Accordingly, the spherical aberrations are corrected.

Figure 9 was obtained from metalenses operating in the visible wavelength region. However, the same concept can be applied to LWIR metalenses. The type-II LWIR metalenses, consisting of Si pillars with a ZnSe antireflection layer and an aperture, demonstrated an ultrawide FOV of 140°.

It should be noted that achieving both high-*NA* values and wide FOVs for metalenses with a hyperbolic phase profile, as described in Equations (1) and (2), remains challenging. The FOV is limited by coma, which is inevitable for oblique incidence and leads to a decrease in focusing efficiency. Therefore, advanced design methods are being investigated, as discussed in Section 8.

## 7. Advanced Functional LWIR Metalenses

One of the key advantages of metalenses is their ability to incorporate advanced functions, such as polarization control, multi-foci, zoom, and reconfigurability. These are the primary attributes of metalenses compared with those of conventional LWIR lens technologies (other than general SWaP-C). This is possible owing to the high degrees of freedom available in the dispersion engineering of meta-atoms, which can be tailored to achieve specific functions. These advanced features are crucial as they enable the development of sophisticated IR cameras for applications such as polarimetric imaging [74], gas analysis, and other analytical purposes [1]. Moreover, analytical IR imaging is expected to become increasingly important following the advancement of artificial intelligence (AI)-based IR imaging technologies [75,76]. The integration of AI can enhance the capabilities of IR imaging systems, making them more efficient and capable of handling complex analytical tasks.

### 7.1. Polarization Control

**Figure 10:**
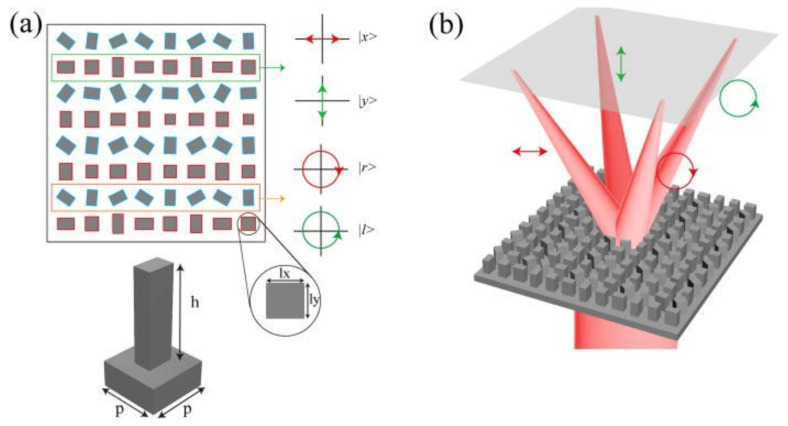
All-Si metalens structure for IR polarimetric imaging. (**a**) Top view of metasurfaces and unit-cell structure. The pillars marked with red and blue outlines are for linear and circular polarizations, respectively. (**b**) Schematic of polarization focusing according to polarization type. Reprinted from [83] with the permission of AIP Publishing.

Polarization plays an important role in analytical IR imaging by enhancing object recognition [77,78]. Polarimetric imaging is typically performed using a polarizer [79,80]. However, incorporating a polarizer as an additional optical component increases the overall cost and can degrade the performance of IR photodetectors owing to unwanted IR radiation emitted by the polarizer. To address this challenge, two main approaches have been proposed, as follows: plasmonic metamaterial absorber-integrated IR photodetectors [81,82], and polarization-selective metalenses [83,84]. Figure 10 shows four distinct types of meta-atoms corresponding to four different types of polarization, demonstrating how polarization control can be inherently designed into metalenses.

As shown in Figure 10a, the meta-atoms with red and blue borders are designed for linear polarizations (x- and y-polarized light) and circular polarizations, respectively. These configurations employ phase modulation controlled by polarization (not to filter) to split and focus LWIR light according to polarization type. As a result, the theoretical efficiency of 50% can be surpassed, enabling chiral imaging. This represents a good example of an advanced LWIR metalens capable of overcoming the performance limitations of conventional IR imaging systems.

### 7.2. Multifocal LWIR Metalenses

**Figure 11:**
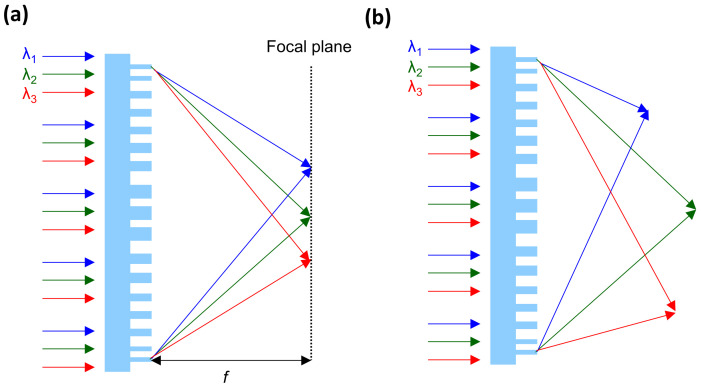
Schematic of multifocal metalenses with different focal positions in the (**a**) same focal plane and (**b**) different focal planes according to the target wavelengths.

As shown in Figure 11, multifocal metalenses can produce different focal positions depending on the target wavelength [59,85,86] and/or polarization [84]. Figure 11a,b show metalenses with the same and different focal lengths, respectively. These two can be realized based on Equations (3) and (4) because the focal length, coordinates, and wavelength can be designed separately and integrated into one metalens.

The multifocal metalens shown in Figure 11a can be applied for spectral analysis using IR image sensors, where different wavelengths are focused in different positions in the same focal plane. Spectrally selective images can, thus, be obtained at different positions within the same image. The multifocal metalens in Figure 11b can be applied to depth sensors by exploiting focal length differences to determine an object’s depth [87]. This functionality shows promise and allows further expansion of the application field of IR image sensors because each pixel in IR image sensors can detect separately each wavelength without a filter [88,89]. Moreover, spatial spectroscopic detection could also be realized with multifocal metalenses. In particular, LWIR multifocal metalenses are promising for gas and biological analysis, as gases and molecules have unique absorption spectra in the LWIR wavelength region.

### 7.3. Zoom Functionality

**Figure 12:**
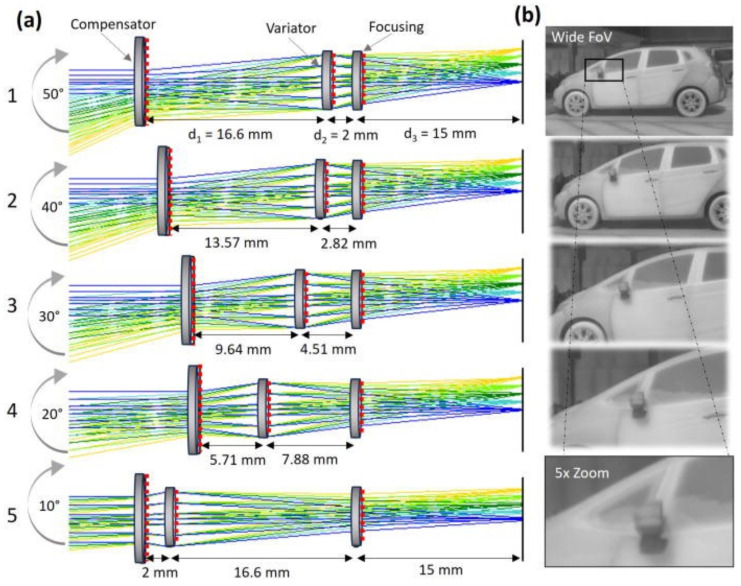
Zoom functionality using triplet metalenses: (**a**) Ray tracing for metalenses configuration. The blue lines correspond to normal angle of incidence rays, while the yellow lines represent angles of incidence of 25° and 5° for the wide and narrow configurations, respectively. The green shading represents the intermediate angles. (**b**) Zoom imaging with an IR camera. Reprinted from [90] with the permission of AIP Publishing.

Zoom functionality is highly important for IR imaging and sensing. However, conventional zoom systems require multiple refractive lenses, resulting in high cost and heavy weight. As shown in Figure 12, triplet metalenses have been used to achieve a zoom range of 5× with a 50° (full) FOV in the widest configuration, and an aperture of 8 mm [90].

This example shows that configuration control among multiple metalenses is a promising strategy for achieving focal length tuning, as demonstrated in the visible wavelength region [91,92,93,94].

### 7.4. Reconfigurable LWIR Metalenses

Reconfigurable or tunable metasurfaces and metalenses are attracting considerable interest to expand the application fields of IR cameras [95]. If properties such as focal length, wavelength, polarization, or phase could be reconfigured, these functionalities would considerably expand the application fields of IR imaging.

The currently used strategy to achieve reconfigurability involves the use of materials that can change their properties through thermal or electrical stimuli. Changes in material constants can control the phase shift of metalenses. For example, thermally tunable metalenses have been demonstrated in the MWIR region using phase-change materials such as VO_2_ [96], Ge_2_Sb_2_Se_4_Te_1_ [97], or thermo-optical polymers [98]. Electrically tunable metalenses have been realized using two-dimensional (2D) materials like graphene [99] in LWIR and 2D ferroelectrics such as CuInP_2_S_6_ in the visible region [100]. Mechanically tunable metalenses using stretchable polydimethylsiloxane substrates have also been demonstrated in the visible wavelength region. It is important to note that while the reference related to graphene [99] describes a Fresnel lens rather than a metalens, the same concept can be readily applied to metalenses.

Although no examples of reconfigurable LWIR metalenses have been reported to date, concepts demonstrated in other wavelength regions can be applied. Given the strong demand for advanced IR imaging, various tunable LWIR metalenses are expected to be proposed, potentially opening up new application fields for IR imaging.

## 8. Challenges

Metalenses have considerable potential not only in the LWIR wavelength region but also in other regions, such as visible, NIR, and MWIR. However, designing metalenses involves addressing complicated trade-offs between key performance characteristics, including achromatism, high *NA*, and wide FOV. As a result, the metalens design requires managing a remarkable number of parameters, making the process complex. Although commercially available software using inverse design methods specialized for metalens design has been launched [101], overcoming trade-offs still requires substantial human effort. In the case of LWIR metalenses, it is required to automate the handling of these numerous parameters using inverse design methods [102] or a topological inverse design method [103]. The term inverse design refers to the use of algorithmic design techniques to design optical structures based on desired functional characteristics [104] and appears to be the most promising approach for commercialization. Recently, AI-related design methods, such as machine and deep learning [105], as well as other techniques like automatic differentiation [106], have been proposed to address these challenges [107,108,109]. Moreover, AI models have also been used for metalens design and high-resolution imaging using metalenses [110]. The progress in this field is rapid, and these demands may be met soon.

Fabrication technology represents another considerable challenge in realizing high-performance LWIR metalenses. The scale of LWIR metalenses is larger than that of visible-wavelength metalenses. As a result, conventional semiconductor fabrication processes can be applied, which reduces fabrication costs. However, the impact of structural fluctuations [111] remains unclear. Structural fluctuations introduced during fabrication—such as scalloping on the sidewalls of pillars and variations in the depth or width of pillars—may affect metalens performance. Therefore, ensuring structural robustness is a critical issue for the successful commercialization of LWIR metalenses. To address this issue, roll-to-plate printing has recently been demonstrated to fabricate metalenses for the visible wavelength [103]. This fabrication method is based on the conventional nano-imprint resin, which is promising for low-cost and high-throughput applications at the nanoscale.

In conventional IR imaging systems, LWIR metalenses are used separately from IR photodetectors. However, recent research has focused on integrating metalenses directly into IR photodetectors, including both quantum- [112,113,114] and thermal-type [115] IR photodetectors. This type of integration holds considerable promise for achieving higher performance, reducing the overall size of IR imaging systems, and lowering product costs.

## 9. Conclusions and Outlook

In this review, we examined all-dielectric metalenses for LWIR imaging applications. Conventional LWIR imaging systems rely on bulky and heavy lenses, posing challenges related to size, weight, and performance. We clarified the limitations of conventional IR lenses and highlighted the advantages of LWIR metalenses in terms of performance and improvements in size, weight, power, and SWaP-C. Key criteria such as achromatism, *NA*, and FOV were discussed, along with their theoretical limits and trade-off relationships based on phase dispersion engineering and meta-atom configurations. Although achieving chromatic LWIR metalenses remains a major challenge, the use of meta-atoms with spatial multiplexing shows potential for addressing this issue. Additionally, we introduced IR images obtained using both chromatic and achromatic LWIR metalenses, demonstrating the promise of LWIR metalenses in practical applications. These achievements suggest that LWIR metalenses possess potential for advancing IR imaging technologies, and continued research in design strategies and fabrication methods will further accelerate their commercialization. Moreover, advanced functional LWIR metalenses and their attributes, including polarization control for IR polarimetric imaging, multifocal, zoom, and reconfigurability, were discussed. These advanced functionalities could broaden the application field of IR imaging because metalenses offer design flexibility to control incident light. The potential of LWIR metalenses to grow rapidly in other applications, such as all-optical diffractive neural networks [116,117] in LWIR, is tremendous because metalenses are expected to realize both higher spatial neuron density and suppression of higher-order diffraction [118]. The design methodology and fabrication tolerance represent the main future challenges. However, these challenges are actively being investigated by many research groups and could be resolved soon. It is hoped that this review will contribute to the development of LWIR metalenses and the expansion of IR imaging applications, such as their integration into mobile phones, by leveraging the unique advantages of LWIR metalenses.

## Figures and Tables

**Table 1:** Comparison of material properties for conventional long-wavelength infrared (LWIR) lens materials.

Materials	Refractive Index at ~10 μm	Dispersion (*υ*_8–12_) ^1^	Density (g/cm^3^)	Thermal Expansion Coefficient (10^−6^/K)	d*n*/d*T* (10^−6^/K)	Knoop Hardness (kgf/mm^2^)
Ge	4.0032 [13]	1700 [14]	5.854 [14]	6 [14]	396 [14]	176 [14]
Si	3.4179 [13]	1863 [15]	2.33 [16]	2.6 [16]	160 [17]	1000 [14]
chalcogenide	2.4–2.8 [18]	30–175 [18]	4.40 [19]	17 [19]	20–103 [18]	109 [14]
ZnS	2.2005 [13]	23 [14]	4.09 [14]	7 [14]	46 [14]	178 [14]
ZnSe	2.4065 [13]	58 [14]	5.27 [14]	7 [14]	57 [14]	137 [14]
CaF_2_	1.3082 [20]	94.99 [21]	3.179 [14]	18.5 [22]	−5.6 [20]	−160 [23]
BaF_2_	1.40133 [24]	81.61 [21]	4.83 [14]	18.1 [22]	−15.45 [24]	82 [14]

^1^ *υ*_8–12_ = (*n*_10_ − 1)/(*n*_8_ − *n*_12_) (*n*_x_ is a refractive index at the wavelength of *x* μm); d*n*/d*T* refers to the thermal coefficient of the refractive index.

**Table 2:** Comparison of product characteristics of conventional LWIR lens materials.

Materials	Processing	Environmental Resistance	Toxicity	Cost
Ge	cutting and polishing	high	nontoxic	high
Si	cutting and polishing	high	nontoxic	low
chalcogenide	press molding	medium	nontoxic ^1^	low
ZnS	press molding	medium	nontoxic ^1^	low
ZnSe	cutting and polishing	medium	toxic	medium
CaF_2_	cutting and polishing	high	nontoxic	low
BaF_2_	cutting and polishing	low	toxic	high

^1^ After vitrification.

## Data Availability

No new data were created or analyzed in this study.
